# Skeletal Deficits in Male and Female down Syndrome Model Mice Arise Independent of Normalized Dyrk1a Expression in Osteoblasts

**DOI:** 10.3390/genes12111729

**Published:** 2021-10-28

**Authors:** Jared R. Thomas, Kourtney Sloan, Kelsey Cave, Joseph M. Wallace, Randall J. Roper

**Affiliations:** 1 Department of Biology, Indiana University-Purdue University Indianapolis, Indianapolis, IN 46202, USA; jt53@iu.edu (J.R.T.); kousloan@iu.edu (K.S.); kacave@iu.edu (K.C.); 2 Department of Biomedical Engineering, Indiana University-Purdue University Indianapolis, Indianapolis, IN 46202, USA; jmwalla@iupui.edu

**Keywords:** DYRK1A, Down syndrome, osteoblasts, sexual dimorphism, gene dosage, skeletal abnormalities, trisomy 21

## Abstract

Trisomy 21 (Ts21) causes alterations in skeletal development resulting in decreased bone mass, shortened stature and weaker bones in individuals with Down syndrome (DS). There is a sexual dimorphism in bone mineral density (BMD) deficits associated with DS with males displaying earlier deficits than females. The relationships between causative trisomic genes, cellular mechanisms, and influence of sex in DS skeletal abnormalities remain unknown. One hypothesis is that the low bone turnover phenotype observed in DS results from attenuated osteoblast function, contributing to impaired trabecular architecture, altered cortical geometry, and decreased mineralization. *DYRK1A*, found in three copies in humans with DS, Ts65Dn, and Dp1Tyb DS model mice, has been implicated in the development of postnatal skeletal phenotypes associated with DS. Reduced copy number of *Dyrk1a* to euploid levels from conception in an otherwise trisomic Ts65Dn mice resulted in a rescue of appendicular bone deficits, suggesting DYRK1A contributes to skeletal development and homeostasis. We hypothesized that reduction of *Dyrk1a* copy number in trisomic osteoblasts would improve cellular function and resultant skeletal structural anomalies in trisomic mice. Female mice with a floxed *Dyrk1a* gene (Ts65Dn,*Dyrk1a*^fl/wt^) were mated with male *Osx-Cre^+^* (expressed in osteoblasts beginning around E13.5) mice, resulting in reduced *Dyrk1a* copy number in mature osteoblasts in Ts65Dn,*Dyrk1a*^+/+/*Osx-Cre*^ P42 male and female trisomic and euploid mice, compared with littermate controls. Male and female Ts65Dn,*Dyrk1a*^+/+/+^ (3 copies of DYRK1A in osteoblasts) and Ts65Dn,*Dyrk1a*^+/+/*Osx-Cre*^ (2 copies of *Dyrk1a* in osteoblasts) displayed similar defects in both trabecular architecture and cortical geometry, with no improvements with reduced *Dyrk1a* in osteoblasts. This suggests that trisomic DYRK1A does not affect osteoblast function in a cell-autonomous manner at or before P42. Although male Dp1Tyb and Ts65Dn mice exhibit similar skeletal deficits at P42 in both trabecular and cortical bone compartments between euploid and trisomic mice, female Ts65Dn mice exhibit significant cortical and trabecular deficits at P42, in contrast to an absence of genotype effect in female Dp1Tyb mice in trabecular bone. Taken together, these data suggest skeletal deficits in DS mouse models and are sex and age dependent, and influenced by strain effects, but are not solely caused by the overexpression of *Dyrk1a* in osteoblasts. Identifying molecular and cellular mechanisms, disrupted by gene dosage imbalance, that are involved in the development of skeletal phenotypes associated with DS could help to design therapies to rescue skeletal deficiencies seen in DS.

## 1. Introduction

### 1.1. Skeletal Abnormalities in DS

Common skeletal phenotypes associated with Down syndrome (DS) include weakened bones, shortened stature and decreased bone mineral density (BMD) as compared to individuals without Ts21 [1,2]. These skeletal deficits are caused by trisomy 21 (Ts21), also the most common cause of congenital cognitive impairment that occurs in about 1/700-1000 live births [3,4,5]. Unlike osteoporosis in individuals without DS, bone disorders in individuals with Ts21 arise due to developmental deficits at critical times of bone mass accretion and may be compounded by subsequent age-related bone loss [6,7,8,9]. Increased average lifespan in individuals with DS [10,11,12] has made the development and advancement of bone disease a concern for older individuals with Ts21 [6,7,8,13,14]. Humans with DS and mouse models of DS exhibit sexual dimorphic features in skeletal anomalies that include age, severity, and skeletal compartment [1,15,16,17,18,19,20]. 

### 1.2. Development of Skeletal Abnormalities in Individuals with DS

Although previous studies have long associated skeletal deficits with DS, it remains unclear how these alterations arise in individuals with Ts21 [1,15,16,17,18,19]. Skeletal phenotypes associated with Ts21 are a consequence of impaired bone formation as exhibited by diminished bone accrual, early attainment of peak bone mass, abnormal mineralization, and low bone mineral density [1,2,13,14,15,17,19,21,22,23,24]. Men and women with DS displayed lower BMD in the femoral neck and lumbar spine much earlier than individuals without DS, with men exhibiting bone loss around 20 years of age, and females experience rapid decline in BMD around 40 years of age [6,7,14]. A heretofore unidentified relationship between increased gene dosage of Hsa21 and perturbation of molecular pathways or cellular functions has been hypothesized [21,24,25,26,27,28,29,30,31]. A report in a woman with DS that quantified the presence and number of bone cells, suggested an adynamic bone phenotype that lacked active osteoclasts [26]. Further investigations of bone phenotypes in humans with DS measured biochemical markers, which revealed that P1NP (marker of bone formation) was reduced in DS without significant differences in CTx (marker of bone resorption) [21]. Low bone mass and BMD phenotypes have been attributed to decreased bone turnover, with an imbalance between bone formation and resorption seen in both humans with DS and DS model mice [21,25,28,32].

### 1.3. Associated Skeletal Abnormalities from DS Mouse Models

Mouse models of DS recapitulate skeletal abnormalities associated with Ts21 that are observed in humans with DS particularly low BMD, early age-related bone loss, and sexual dimorphism [14,15,20,25,28,33,34]. Male Ts65Dn trisomic model mice at 6 and 16 weeks have significantly decreased BMD, disorganized trabecular architecture, perturbed cortical geometry, and reduced bone strength compared to euploid animals [33]. At 6 weeks of age, there were decreased osteoblast and increased osteoclast activity and decreased bone formation rate in male Ts65Dn mice. At 12 weeks of age, male Ts65Dn mouse femurs had significantly lower bone mass and mechanical strength, decreased osteoblast and osteoclast development, and reduced bone formation rate [25]. Three copies of genes orthologous to Hsa21 in mice are thought to alter bone homeostasis, and bone biomarkers related to resorption (TRAP) and formation (P1NP) were not different in 3-month-old male Ts65Dn mice, but were significantly decreased at 24 months in Ts65Dn as compared to euploid mice [25]. Discrepancies between Fowler et al. and Blazek et al. likely result from different stages of maturity (6 weeks vs. 12 weeks), suggesting trisomic *Dyrk1a* may play a dynamic role in bone remodeling and regulation of osteogenic cell types.

The Dp1Tyb DS mouse model, containing three copies of all Mmu16 genes orthologous to Hsa21, displayed sexually dimorphic skeletal deficits associated with Ts21 [20,35]. At 6 weeks of age, Dp1Tyb male animals displayed reduced trabecular bone organization and impaired cortical architecture compared to euploid controls, however female Dp1Tyb mice did not show significant differences in the trabecular compartment. Overall, there were significant differences between male and female Dp1Tyb mice, with female mice exhibiting lower bone measures compared to male mice. At 16 weeks of age, cortical deficits were apparent in both male and female Dp1Tyb as compared to euploid littermate mice. It is hypothesized that skeletal differences found in individuals with DS and DS mouse models are different between males and females and may be due to changes in number and/or function of osteoblasts and/or osteoclasts. Based on human and mouse studies, it is hypothesized that a low bone turnover phenotype in DS results from impaired cellular function or dysregulated molecular signaling.

### 1.4. DYRK1A Gene Dosage Contributes to the Development of Skeletal Phenotypes in Ts21

*DYRK1A*, a gene found in three copies of most DS mouse models and individuals with DS, is a candidate gene for cognitive impairment, Alzheimer-like phenotypes, and skeletal abnormalities related to DS [36,37,38,39,40]. Transgenic mice overexpressing *Dyrk1a* displayed appendicular skeletal deficiencies and osteopenia [27]. *Dyrk1a* is considered a candidate gene for skeletal abnormalities seen in humans with DS and Ts65Dn model mice [30,31,33]. Returning *Dyrk1a* copy number to normal levels in an otherwise trisomic animal led to a rescue of skeletal deficits, improving both cortical and trabecular architecture in 6-week-old Ts65Dn male mice [31]. Six-week-old Ts65Dn trisomic animals with two copies of *Dyrk1a* displayed improved bone formation, increased osteoblast activity and decreased osteoclast number and activity compared the Ts65Dn littermate controls with three copies of *Dyrk1a*. 

### 1.5. Hypothesis; Reduction of Dyrk1a in Osteoblasts Will Improve Skeletal Deficits Associated with Ts21 in Ts65Dn Male and Female Mice

Not much is known about specific causes of skeletal abnormalities in DS, and more information is needed to understand when these deficits appear, possible genes related to altered skeletal development, and how skeletal phenotypes present differently in males and females with DS [29]. Based on previous work, we proposed that *Dyrk1a* over-expression at or before 6 weeks would cause attenuated osteoblast function, leading to reduced bone formation and low bone mass compared to euploid control animals [31]. We hypothesized that reduction of *Dyrk1a* copy number specifically in osteoblasts would improve deficits associated with low bone mineral density and bone formation in trisomic Ts65Dn male and female mice. Additionally, we proposed that male and female mice would present with sexually dimorphic skeletal phenotypes, like those found in Dp1Tyb DS mouse model, as this is the first time female Ts65Dn mouse skeletal phenotypes have been characterized. To investigate the role of trisomic *Dyrk1a* in osteoblast bone formation, we established a mouse model in which one copy of *Dyrk1a* was conditionally deleted in osteoblast lineage cells using an *Osterix* (*Osx/Sp7*) promoter driven Cre recombinase. 

## 2. Materials and Methods

### 2.1. Animals

Female B6EiC3Sn a/A-Ts(1716)65Dn (Ts65Dn—stock number 001924) and male B6C3F1 mice were purchased from the Jackson Laboratory (Bar Harbor, ME, USA). Ts65Dn offspring were generated at Indiana University-Purdue University Indianapolis (IUPUI) by crossing Ts65Dn females (small marker (trisomic) chromosome) with B6C3F1 males, with resultant offspring having an approximate 50% C57BL/6 and 50% C3H/HeJ background. Approximately every 6 months, new Ts65Dn and B6C3F1 mice were purchased from the Jackson Laboratories and added to the colony. Mice with lox sites flanking exons 5 and 6 of *Dyrk1a* (C57BL/6-*Dyrk1a*^tm1Jdc^ or *Dyrk1a*^fl/fl^) on a C57BL/6 background were obtained from Dr. John Crispino [41] and were mated to C3H/HeJ mice. The resultant B6C3F1.*Dyrk1a*^fl/wt^ mice were intercrossed and bred to create homozygous B6C3.*Dyrk1a*^fl/fl^ mice through genotyping and test cross mating to mimic the genetic background of Ts65Dn mice. *Osx1*-GFP::Cre mice ((B6.Cg-Tg(Sp7-tTA,tetO-EGFP/cre)1Amc/J *or Osx*-*Cre*) were obtained from Jackson Laboratories and crossed to C3H/HeJ mice. The resulting B6C3F1 *Osx*-*Cre* mice were intercrossed, and homozygous B6C3.*Osx*-*Cre* mice (similar genetic background as Ts65Dn mice) were generated and identified through genotyping. 

Female Ts65Dn and male *Dyrk1a*^fl/fl^ mice were bred to generate Ts65Dn,*Dyrk1a*^fl/wt^ animals. Ts65Dn,*Dyrk1a*^fl/wt^ females were crossed to *Dyrk1a*^fl/fl^ males to produce homozygous Ts65Dn,*Dyrk1a*^fl/fl^ mice. However, out of 51 litters (males *n* = 95; females *n* = 98) we only achieved heterozygous Ts65Dn,*Dyrk1a*^fl/wt^, mice with only one floxed allele, indicating that there was selection against homozygous Ts65Dn, *Dyrk1a*^fl/fl^ mice. We anticipate that this negative selection occurred prenatally, as we did not notice dead perinatal mice from these litters before weaning. 

To generate Ts65Dn mice with a functional reduction of the kinase domain in one of the *Dyrk1a* alleles in mature osteoblasts, Ts65Dn,*Dyrk1a*^fl/wt^ females were bred to homozygous B6C3.*Osx*-*Cre* males. Mice with the following genotypes were generated: 3 copies of *Dyrk1a* in osteoblasts (Ts65Dn,*Dyrk1a*^+/+/+^ (same as Ts65Dn)) males *n* = 10, females *n* = 12; trisomic mice with 2 active copies of *Dyrk1a* in osteoblasts (Ts65Dn,*Dyrk1a*^+/+/*Osx-Cre*^ (same as *Dyrk1a*^fl/wt^,*Osx-Cre*+)) males *n* = 5, females *n* = 10; euploid mice with 2 copies of *Dyrk1a* in osteoblasts (Eu,*Dyrk1a^+/+^*) males *n* = 14, females *n* = 29; euploid mice with 1 active copy of *Dyrk1a* in osteoblasts (Eu,*Dyrk1a*^+/*Osx-Cre*^ (same as *Dyrk1a*^fl/wt^, *Osx-Cr*e+ ) males *n* = 7, females *n* = 15. We prioritized using litters (*n* = 16) with at least one Ts65Dn,*Dyrk1a*^+/+/*Osx-Cre*^ mouse in the litter for the analyses. Next we included litters (*n* = 8) with at least 1 Eu,*Dyrk1a*^+/*Osx-Cre*^ mouse, and then litters (*n* = 4) with at least 1 Ts65Dn,*Dyrk1a*^+/+/+^ mouse. Offspring were euthanized at postnatal day 42 (P42), animals were weighed, and femurs dissected. Left femurs were stored in PBS-soaked gauze and used for Microcomputed tomography (µCT) analysis and mechanical testing. All animal use and protocols were approved by the Institutional Animal Care & Use Committee (IACUC) at the IUPUI School of Science (SC255R and SC298R) and adhered to the requirements in the NIH Guide for the Care and Use of Laboratory Animals.

### 2.2. Genotyping

Ts65Dn mice were genotyped using the breakpoint PCR protocol [42]. To detect the presence of the loxP sites flanking exons 5 and 6 of Dyrk1a, PCR utilizing primers 25,066 (forward) (TACCTGGAGAAGAGGGCAAG) and 25,067 (reverse) (GGCATAACTTGCATACAGTGG) as previously done [41] or using primers Cond DyrkF (forward) (ATTACCTGGAGAAGAGGGAAG) and Cond DyrkR (reverse) (TTCTTATGACTGGAATCGCC); PCR conditions 3 min at 95 °C, (20 s at 95 °C, 30 s at 53 °C, 1 min and 30 s at 72 °C) for 40 cycles. 

To genotype for *Osx-Cre*, primers 30,295 (forward) (GAGAATAGGAACTTCGGAATAGTAAC) and 30,296 (reverse) (CCCTGGAAGTGACTAGCATTG) were used that amplify a 198 bp band (https://www.jax.org/Protocol?stockNumber=006361&protocolID=20366, accessed on 15 August 2021) or primers were used Osx10 (forward) (CTCTTCATGAGGAGGACCCT) and TGCK (reverse) (GCCAGGCAGGTGCCTGGACAT) with a ~500 bp product; PCR conditions: 1 min at 94 °C, (30 s at 94 °C, 30 s at 55 °C, 30 s at 68 °C, 2 min at 72 °C) for 39 cycles [43]. Primers for internal controls were consistent throughout the study 25,775 (forward) (AGAGAGCTCCCCTCAATTATGT) and 25,776 (reverse) (AGCCACTTCTAGCACAAAGAACT) amplifying a 235 bp product. 

All Ts65Dn,*Dyrk1a*^+/+/*Osx-Cre*^ and Eu,*Dyrk1a*^+/*Osx-Cre*^ mice were genotyped to detect deletion of the floxed *Dyrk1a* allele, using primers DOXF (forward) (ACCTGGAGAAGAGGGCAAGA) and DOXR (reverse) (GCCACTGTGTGAGGAGTCTT) to amplify a 214bp product; PCR conditions 3 min 95 °C, (20 se at 95 °C, 20 s at 60 °C, 30 s at 72 °C) for 35 cycles. 

### 2.3. Microcomputed Tomography (µCT)

Left femurs were thawed to room temperature and scanned using high-resolution µCT system, SkyScan 1172 µCT (SkyScan, Kontich, Belgium) using the following parameters: voltage 60 kV, resolution 12 µM, using a filter Al 0.5 mm, with a 0.7° angle increment and four frames average. Calibrations were performed daily using two cylindrical hydroxyapatite phantoms (0.25 and 0.75 g/cm^3^ calcium hydroxyapatite). Femurs were rewrapped in PBS-soaked gauze and stored at −20 °C until mechanical testing. Reconstruction analysis was performed using NRecon and CTan software (Skyscan, Bruker µCT, Belgium). Trabecular microarchitecture and cortical geometry were accomplished using previously published protocol [30,44]. 

Trabecular analysis was performed on the distal metaphysis with a region of interest (ROI) defined as 10% of the total bone length, beginning at the distal growth plate and extending proximally approximately 1mm. The ROI was auto-segmented using custom Matlab (MathWorks, Inc. Natick, MA, USA) code [44]. Measurements from trabecular architecture (bone volume fraction (BV/TV), trabecular thickness, number, and separation), bone mineral density (BMD) and tissue mineral density (TMD) were obtained using CTAn.

Cortical analysis was performed at a standard site 60% of the total bone length away from the distal growth plate. Seven transverse slices were generated from the standard site and cortical geometric properties (total cross-sectional area, cortical thickness, marrow arrow, cortical area, periosteal bone surface and endosteal bone surface) were obtained from using a custom Matlab code. 

### 2.4. Mechanical Testing 

The mechanical properties of the femur were determined using a mechanical testing machine (TA ElectroForce 3200; Eden Prairie, MN, USA and TestResources, Shakopee, MN, USA). The femurs were thawed to room temperature and tested in 3 point bending the anterior-posterior direction with the posterior surface in compression (7 mm support span). The load was applied to the midpoint of each bone. The femur was preloaded using 0.5 N to establish contact with the bone and then monotonically tested to failure at a displacement rate of 0.025 mm/s while fully hydrated. Load and deflection were recorded, from which structural strength (yield and ultimate), stiffness (slope of the linear portion of the force versus displacement curve), and deformation (yield deformation, postyield deformation, and total deformation) were determined [45,46]. 

Along with the force/displacement data, the moment of inertia about the axis of bending (I_ML_) and distance from the centroid to the tensile surface of the bone in tension derived from μCT data described from above and were used to map load-displacement (structural-level properties) into stress vs. strain curves (predicted tissue-level properties) using standard equations derived from Euler-Bernoulli beam theory for three point bending [45]. The tissue level properties were determined from the stress vs. strain curve. The yield point was calculated using the 0.2% offset method based on the stress-strain curve. The modulus of elasticity was calculated as the slope of the linear portion of the stress-strain curve.

### 2.5. Statistical Analysis

The assumptions of normality and homogeneity of variance were assessed and there were no significant violations. A two-way factorial ANOVA was performed on µCT and mechanical testing data to determine potential main effects of genotype, sex, and their interaction grouping male and female Ts65Dn,*Dyrk1a*^+/+/+^ and Ts65Dn,*Dyrk1a*^+/+/*Osx-Cre*^, as well as Eu,*Dyrk1a*^+/+^ and Eu,*Dyrk1a*^+/*Osx-Cre*^ mice together. Male and female groups of mice were then analyzed separately by a posthoc two-way ANOVA to detect potential sex-specific effects of ploidy (trisomic vs. euploid) and *Dyrk1a* copy number. For each parameter with a significant interaction, posthoc analyses (Tukey’s multiple comparisons test) determined differences between groups. Chi-squared goodness of fit test was performed to determine statistical differences in expected and observed genotype ratios in male and female Ts65Dn animals. 

## 3. Results

### 3.1. Suspected Perinatal Death of Ts65Dn,Dyrk1a^+/+/Osx-Cre^ Male Mice

From (Ts65Dn,*Dyrk1a*^fl/wt^ × B6C3.*Osx*-*Cre*) matings we expected one in every eight pups to be Ts65Dn,*Dyrk1a*^+/+/*Osx-Cre*^ (two active copies of *Dyrk1a*) and Eu,*Dyrk1a*^+/*Osx-Cre*^ (one active copy of *Dyrk1a*), but we noticed fewer male Ts65Dn,*Dyrk1a*^+/+/*Osx-Cre*^ and Eu,*Dyrk1a*^+/*Osx-Cre*^ mice than expected. While beginning our (Ts65Dn,*Dyrk1a*^fl/wt^ × B6C3.*Osx*-*Cre*) matings, we also found 44 dead pups in their home cage including 10 Eu,*Dyrk1a*^+/+^, 4 Eu,*Dyrk1a*^+/*Osx-Cre*^, 11 Ts65Dn,*Dyrk1a*^+/+/+^, 3 Ts65Dn,*Dyrk1a*^+/+/*Osx-Cre*^ male; and 7 Eu,*Dyrk1a*^+/+^, 0 Eu,*Dyrk1a*^+/*Osx-Cre*^, 8 Ts65Dn,*Dyrk1a*^+/+/+^, 1 Ts65Dn,*Dyrk1a*^+/+/*Osx-Cre*^ female mice. We performed a Chi-square goodness of fit test and concluded that ratios of the dead animals were consistent with expected outcomes and that significant selection against male Ts65Dn,*Dyrk1a*^+/+/*Osx-Cre*^ and Eu,*Dyrk1a*^+/*Osx-Cre*^ mice after birth did not occur, although there appeared to be fewer male Ts65Dn,*Dyrk1a*^+/+/*Osx-Cre*^ and Eu,*Dyrk1a*^+/*Osx-Cre*^ mice generated. 

### 3.2. Trabecular Deficits in Female and Trisomic Mice

**Figure 1:**
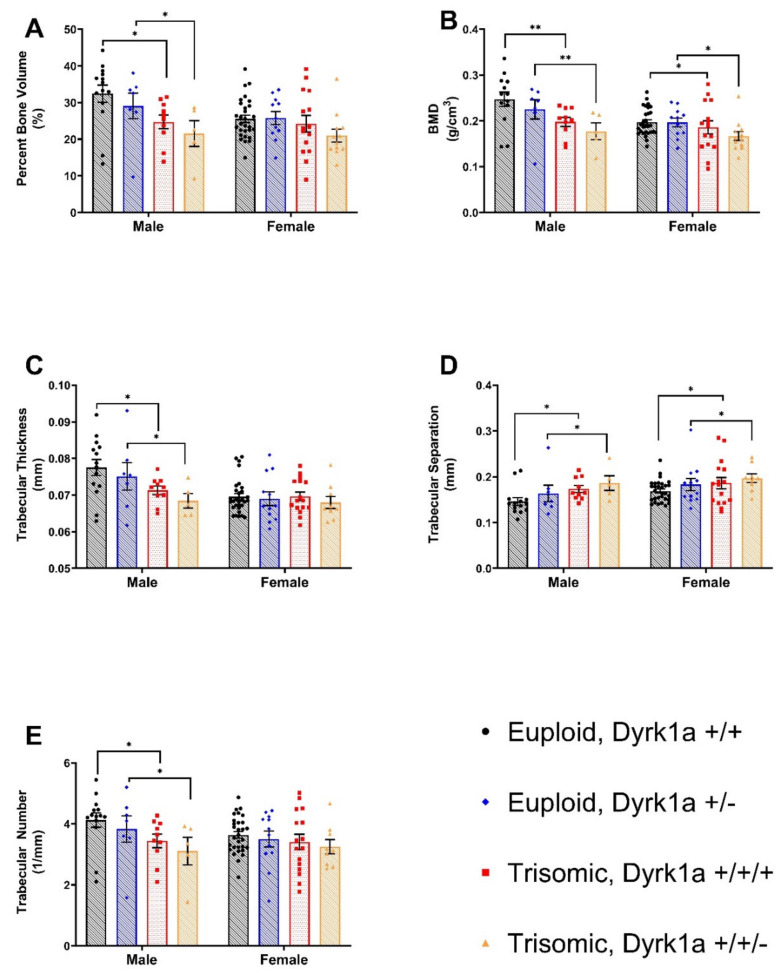
Trabecular architecture differs between male and female Euploid and Ts65Dn animals at six weeks of age (**B**–**D**). (**A**) Percent Bone Volume (BV/TV); Main effect of ploidy for male and female. (**B**) Bone mineral density (BMD); Main effect ploidy for male and female. (**C**) Trabecular Thickness (Tb.Th) Main effect of ploidy for male mice. (**D**) Trabecular separation; Main effect of ploidy for male and female. (**E**) Trabecular Number (Tb.N) Main effect of ploidy for male mice. Mean ± SD; bars between groups of mice denote significance; *p*-value 0.1234 (ns); 0.0332 (*); 0.0021 (**).;.

Eight groups were generated to test our hypotheses: Male and female Ts65Dn,*Dyrk1a*^+/+/+^, Ts65Dn,*Dyrk1a*^+/+/*Osx-Cre*^, Eu,*Dyrk1a*^+/+^, and Eu,*Dyrk1a*^+/*Osx-Cre*^ mice. When trabecular properties in these eight groups were compared together, male mice as compared to female mice had a higher bone mineral density (BMD) (*p* = 0.0079), trabecular thickness (Tb.Th) (*p* = 0.0028), and a lower trabecular separation (Tb.Sp) (*p* = 0.0397) (sex effect). All euploid as compared to trisomic mice displayed a higher BMD (*p* = 0.0010), BV/TV (*p* = 0.0033), Tb.Th (*p* = 0.0283), trabecular number (Tb.N) (*p* = 0.0361) and lower Tb.Sp (0.0113) (ploidy effect). There were no interactive effects observed between sex and ploidy (Figure 1).

When only male Ts65Dn,*Dyrk1a^+/+/+^*, Ts65Dn,*Dyrk1a*^+/+/*Osx-Cre*^, Eu,*Dyrk1a^+/+^*, and Eu,*Dyrk1a*^+/*Osx-Cre*^ mice were compared for the effects of the extra chromosomal material (ploidy) and the effects of *Dyrk1a* copy number in osteoblasts (*Dyrk1a*), euploid mice had stronger trabecular parameters than trisomic mice for BMD (*p* = 0.009), BV/TV (*p* = 0.0127), Tb.Th (*p* = 0.018), Tb.N (*p* = 0.0386), and Tb.Sp (*p* = 0.0451), consistent with previous studies in Ts65Dn and Dp1Tyb male DS mouse models [20,33] (Figure 1). There was no significant difference between trisomic male mice with 2 or 3 functional copies of *Dyrk1a* in the osteoblasts. 

When female mice were analyzed independent of males, trabecular properties were better in euploid as compared to trisomic mice for BMD (*p* = 0.0425), Tb.Sp (*p* = 0.0121) but not BV/TV (*p* = 0.0786), Tb.Th (*p* = 0.2631) or Tb.N (*p* = 0.0552) (Figure 1). This is the first time that trabecular bone has been quantified in female Ts65Dn mice; we found that female Ts65Dn as compared to control mice had significantly reduced/altered trabecular architecture/properties at 6 weeks of age. These findings differ from no significant trabecular differences identified at 6 weeks of age between Dp1Tyb and euploid female DS model mice [20]. In the analysis of female mice, like male littermates, there was no significant effect of reduced *Dyrk1a* copy number in the osteoblasts. 

### 3.3. Skeletal Alterations in Cortical Architecture in Trisomic Mice

**Figure 2:**
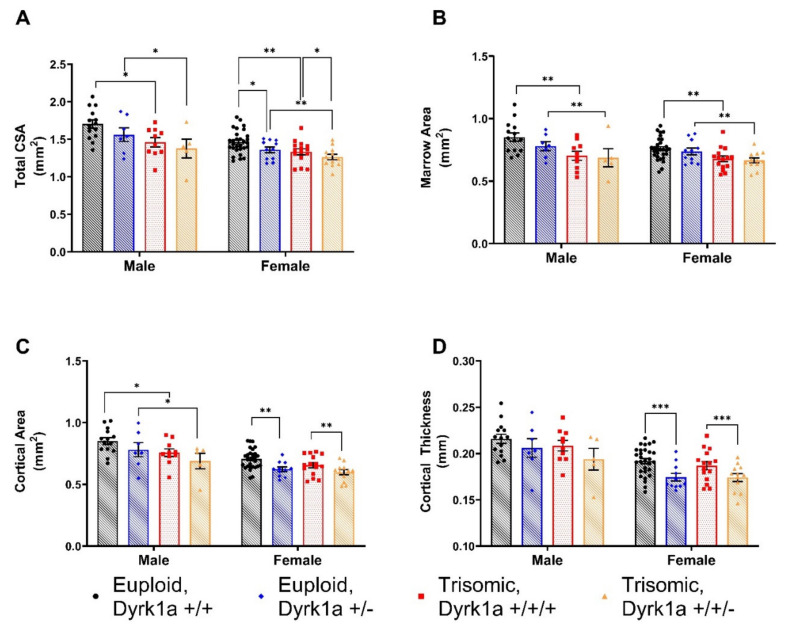
Cortical bone parameters are significant different between male and female Euploid and Ts65Dn animals (**A**–**D**). (**A**) Total cross-sectional area (CSA) main of effect of ploidy in males and main effect of ploidy and *Dyrk1a* genotype in females. (**B**) Marrow Area (Ma.Ar); main effect of ploidy in male and female animals. (**C**) Cortical Area (Ct.Ar); main effect of ploidy in male mice; main effect of *Dyrk1a* copy number in female. (**D**) Cortical Thickness (Ct.Th); main effect of *Dyrk1a* copy number in female animals. Mean ± SD; bars between groups of mice denote significance; *p*-value 0.1234 (ns); 0.0332 (*); 0.0021 (**); 0.0002 (***).

**Figure 3:**
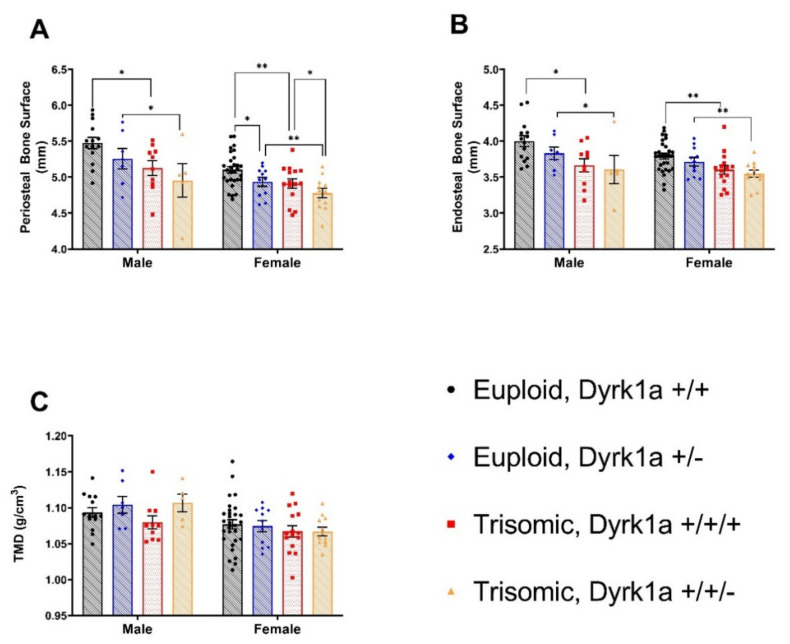
Cortical bone analysis revealed significant differences between male and female Euploid and Ts65Dn animals (**A**–**C**). (**A**) Periosteal Bone Surface (Ps.BS); main effect of ploidy in male and female mice; main effect of *Dyrk1a* genotype in female mice. (**B**) Endosteal Bone Surface (Es.BS); main effect of ploidy in male and female mice. (**C**) No significant differences between ploidy or *Dyrk1a* genotype. Mean ± SD; bars between groups of mice denote significance; *p*-value 0.1234 (ns); 0.0332 (*); 0.0021 (**).

When cortical skeletal microarchitecture was examined in all eight groups with males and females together, there were both a sex and a ploidy effect (with no interaction), with males showing greater cortical properties in total cross-sectional area (CSA) (*p* < 0.0001), marrow area (Ma.Ar) (*p* = 0.0428), cortical area (Ct.Ar) (*p* < 0.0001), cortical thickness (Ct.Th) (*p* < 0.0001) and periosteal (Ps.BS) (*p* < 0.0001), endosteal bone surfaces (Es.BS) (*p* = 0.0452), and tissue mineral density (TMD) (*p* = 0.0003) **(**Figure 2 and Figure 3). Euploid mice displayed greater total CSA (*p* < 0.0001), Ma.Ar (*p* < 0.0001), Ct.Ar (*p* < 0.0001), Ct.Th (*p* = 0.0019), Ps.BS (*p* < 0.0001), and Es.BS (*p* < 0.0001) but not TMD (*p* = 0.2958) compared to trisomic mice. 

When males were analyzed separately, male euploid mice had significantly greater total CSA (*p* = 0.0104), Ma.Ar (*p* = 0.0094), Ct.Ar (*p* = 0.0341), Ps.BS (*p* = 0.0149) and Es.BS (*p* = 0.0144) compared to male trisomic mice (Figure 2 and Figure 3). There was no significant improvement in Ts65Dn,*Dyrk1a*^+/+/*Osx-Cre*^ male mice compared to Ts65Dn,*Dyrk1a^+/+/+^* male mice for cortical microarchitecture. There was a trend toward an increase in TMD in male mice (*p* = 0.0645) with reduced *Dyrk1a* copy number in osteoblasts.

Similarly, female euploid mice had significantly greater total CSA (*p* = 0.0041), Ma.Ar (*p* = 0.0014), Ps.BS (*p* = 0.0059), and Es.BS (*p* = 0.0022) than female trisomic mice. In female animals, there was a main effect of *Dyrk1a* normalization in total CSA (*p* = 0.0261), Ct.Ar (*p* = 0.0009), Ct.Th (*p* = 0.0004), and Ps.BS (*p* = 0.0161). Normalization of *Dyrk1a* did not improve cortical measurements in female Ts65Dn,*Dyrk1a*^+/+/*Osx-Cre*^ animals, as hypothesized (Figure 2 and Figure 3). Rather, reduced *Dyrk1a* copy number in both female euploid and Ts65Dn mice showed a significant decrease in some cortical parameters measured, with the exceptions of Es.BS (*p* = 0.2378), Ma.Ar (*p* = 0.4673) and TMD (*p* = 0.8694). There was no interaction between ploidy and *Dyrk1a* copy number in osteoblasts. 

### 3.4. Dyrk1a Overexpression in Osteoblasts Does Not Impair Whole Bone or Material Properties as Demonstrated by Mechanical Testing

#### 3.4.1. Whole Bone Mechanical Properties

When males and females were analyzed together, there were significant differences in mechanical whole bone properties affected by sex and ploidy. Female as compared to male mice had significantly decreased yield force (*p* = 0.0474), and ultimate force (*p* = 0.0003); this suggests that female mice had reduced resistance to elastic deformation and decreased whole bone strength. Ts65Dn as compared to euploid control femurs had a significant decrease in yield force (*p* = 0.004), displacement to yield (*p* < 0.0001), postyield displacement (*p* = 0.005), and total displacement (*p* = 0.0278). Taken together, these data suggest that Ts65Dn mice reach yield at a lower displacement and lower ductility as compared with control mice. Normalization of *Dyrk1a* copy number did not rescue trisomic deficits in whole bone properties, furthermore Eu,*Dyrk1a*^+/*Osx-Cre*^ animals did not exhibit significant impairments compared to Eu,*Dyrk1a*^+/+^ or Ts65Dn,*Dyrk1a*^+/+/+^ animals. 

When male mice were analyzed separately, euploid mice showed significantly better yield force (*p* = 0.0471) and ultimate force (*p* = 0.0267) as compared to trisomic mice, and there were no effects of reduced *Dyrk1a* copy number in the osteoblasts in male mice or interactive effects. When only female mice were analyzed separately, there were interactive effects of ploidy and *Dyrk1a* copy number in ultimate force (*p* = 0.0035), displacement yield (*p* = 0.0149), and stiffness (*p* = 0.0001). Female euploid as compared to trisomic mice showed increased yield force (*p* = 0.0455), ultimate force, displacement to yield, and stiffness. This suggests female Ts65Dn mice had reduced strength and resistance to permanent deformation/fracture compared to euploid controls (Table 1 and Table 2).

#### 3.4.2. Material Level Properties

When analyzed together (males and females), resilience (*p* < 0.0001) and toughness (*p* = 0.0032) were significantly reduced in trisomic as compared to euploid mice, suggesting trisomic femurs required less energy before permanent deformation. Female mice as compared to male mice had lower resilience (*p* = 0.0323). With males and females analyzed together, there was also a significant effect of ploidy for yield stress (*p* = 0.0374), strain to yield (*p* < 0.0001), and total strain (*p* = 0.005), with Ts65Dn,*Dyrk1a*^+/+/+^ lower than Eu,*Dyrk1a*^+/+^ mice. This suggests Ts65Dn,*Dyrk1a*^+/+/+^ femurs were less elastic than Eu,*Dyrk1a*^+/+^ controls and showed impaired ability to resist deformation. 

Male mice analyzed separately to determine differences between ploidy and *Dyrk1a* copy number in osteoblasts revealed no difference between trisomic and euploid animals. When female mice were analyzed separately, female trisomic mice had lower total strain (*p* = 0.0259) compared to euploid littermates (Table 1 and Table 2).

## 4. Discussion

Skeletal deficits associated with Ts21 are most likely the result of impaired skeletal development and bone homeostasis. Skeletal abnormalities in individuals with DS appear to occur in males before females, yet how developmental abnormalities during adolescent stages in males and females affect later bone homeostasis abnormalities are unknown. Using DS mouse models allows for examination of how sex, age, and genetic composition affect DS-related skeletal phenotypes. Here we report the first examination of skeletal phenotypes in female Ts65Dn mice at six weeks of age, during typical time of sexual maturity and bone accrual. We also examined the effect of *Dyrk1a* copy number in osteoblast progenitors in 6-week-old male and female Ts65Dn and euploid littermate mice. 

### 4.1. Bone Deficits in Ts65Dn Female Mice

At 6 weeks of age, trabecular microarchitecture was significantly worse in all female as compared to male mice, and trisomic female as compared to euploid female littermate mice. In contrast to no significant trabecular bone deficits in Dp1Tyb female mice at 6 weeks of age [20], female six-week-old Ts65Dn mice exhibited trabecular bone deficits as compared to female littermate control mice. Cortical geometry was significantly different between male and female mice, with female mice having lower total CSA, Ma.Ar, Ct.Th, Ps.BS, Es.BS, and TMD compared to male mice. Also, female Ts65Dn mice had deficits in cortical microarchitecture compared to female euploid littermate mice. This observation was consistent with the cortical deficits seen in Dp1Tyb female mice indicating that three copy genes present in both Ts65Dn and Dp1Tyb mice contribute to cortical skeletal phenotypes. Lastly, there was a significant sex effect for bone and material properties between male and female Ts65Dn mice. Male mice had greater yield force, ultimate force, and resilience compared to female mice, indicating male mice had relatively stronger bones compared to female animals. Female trisomic as compared to control mice had diminished whole bone and material properties. Taken together, these data indicate that both 6-week-old Ts65Dn male and female mice have significant changes in trabecular, cortical, and mechanical bone properties. 

### 4.2. Effects of Dyrk1a Copy Number Reduction in Osteoblasts

Previous studies have identified that three copies of *Dyrk1a* are sufficient for postnatal establishment and maintenance of abnormal adolescent male Ts65Dn skeletal phenotypes [31]. We hypothesized that the loss of osteoblast number and activity during skeletal maturation in DS model mice was significantly associated with developmental skeletal deficits found in both male and female Ts65Dn mice. We further hypothesized that a reduction of *Dyrk1a* copy number in osteoblasts of trisomic mice would correct DS-associated bone deficits. Differences in *Dyrk1a* functional copy number in osteoblasts did not affect the trabecular compartment of either female or male mice. In female mice, there was a significant main effect of *Dyrk1a* functional copy number for CSA, Ct.Ar, Ct.Th, and Ps.BS, for both euploid and trisomic animals; however, the reduction of *Dyrk1a* copy number resulted in impaired, not improved, cortical geometry in both trisomic and euploid mice. 

Male mice also showed somewhat of a decrease in cortical parameters when *Dyrk1a* copy number was reduced in osteoblasts. Reduced *Dyrk1a* copy number in the osteoblasts led to altered cortical geometry, possibly indicating a relationship between *Dyrk1a* activity related to the osteoblasts and skeletal development. Although male as compared to female mice had a significantly greater TMD, no differences in TMD were seen in female mice. A trend toward an increase in TMD in male mice with reduced *Dyrk1a* copy number in osteoblasts suggested that *Dyrk1a* in osteoblasts may contribute to mineralization at the mid-diaphysis. 

### 4.3. Potential Mechanisms for Dyrk1a Copy Number in Ts65Dn Mice

Lack of improvement of skeletal phenotypes in Ts65Dn,*Dyrk1a*^+/+/*Osx-Cre*^ animals suggests *Dyrk1a* skeletal deficits arise due to synergistically disrupted molecular or cellular mechanisms involved in skeletal development. The global reduction of *Dyrk1a* improved skeletal abnormalities in 6-week male Ts65Dn mice, suggesting that global reduction of *Dyrk1a* affects multiple cell types during development and most likely affecting regulation of skeletal development and maintenance in a non-autonomous effect. Instead of just affecting osteoblasts, trisomic *Dyrk1a* may alter the balance between bone resorption and formation leading to osteopenic phenotypes seen in Ts65Dn animals. *Dyrk1a* targets multiple biological process and signaling pathways such as transcription, mRNA splicing, cell cycle division and differentiation. DS skeletal abnormalities associated with *Dyrk1a* overexpression target various cytokines, growth factors, cell-cell communication, and interactions with extracellular matrices that lead to altered skeletal development. Moreover, genetic reduction of *Dyrk1a* in other cell types, besides just osteoblasts, could be responsible for the amelioration of skeletal abnormalities. DYRK1A is a negative regulator of NFAT and RCAN1 transcription factors [27,47]. DYRK1A and RCAN1 (both located on Hsa21) overexpression negatively regulates NFAT transcriptional activity [27,48]. NFAT is known as the master regulator of osteoclastogenesis and plays a role in osteoblast differentiation [49]. RCAN1 is a negative regulator of osteoclast differentiation by binding to calcineurin and inhibits NFAT transcriptional activity, its overexpression can lead to attenuated osteoclast differentiation [27,50,51]. *Dyrk1a* has been known to regulate Hedgehog signaling (Hh), which has been implicated in bone formation and limb formation [52,53,54]. *Dyrk1a* overexpression may affect osteoblast differentiation or function of precursor osteoblastic cells upstream of Osx expression by altering regulators involved in osteoblast differentiation like Ihh, Runx2, or Wnt. Runx2 is essential for osteoblast differentiation and required for Osx expression [55,56]. Activation of Runx2 directs mesenchymal stem cells into preosteoblasts, and they undergo differentiation into mature osteoblasts by expressing specific molecular markers [57]. Pre-osteoblasts are thought to provide maintenance of the extracellular matrix and express ALP and osteocalcin, components of collagen production [57]. It is possible pre-osteoblast function or skeletal development may be compromised before *Dyrk1a* normalization [52,55,56,58].

### 4.4. Limitations: Potential Uncharacterized Effects of DYRK1A and Low Sample Size of Male Ts65Dn,Dyrk1a^+/+/Osx-Cre^ on Differences Skeletal Parameters

We used PCR to confirm the genetic reduction of a single copy of *Dyrk1a* in mature osteocytes, but because of design study and low numbers of osteocytes in bone, we were unable to confirm a quantitative reduction of DYRK1A in these mice. Additionally, epigenetic mechanisms affecting gene transcription might mask or balance the effects of supernumerary genes in osteoblasts and the downstream effects on bone phenotypes, and these potential confounders were not accounted for in this experimental design. We noted reduced sample sizes for both male Ts65Dn,*Dyrk1a*^+/+/*Osx-Cre*^ and Eu,*Dyrk1a*^+/*Osx-Cre*^ mice as compared to their female counterparts. Though the differences in numbers of male mice were not statistically significant, we believe this paucity of male mice with one fewer copy of *Dyrk1a* in their osteoblasts affected the results of our experiments, including the ability to detect differences between males and females in the interactive effects between ploidy and *Dyrk1a* copy number in the cortical and mechanical studies. Furthermore, we noticed lack of differences between *Dyrk1a* genotype in male mice in mechanical properties possibly the result of lower sample sizes in Eu,*Dyrk1a*^+/*Osx-Cre*^ and Ts65Dn,*Dyrk1a*^+/+/*Osx-Cre*^ mice. We hypothesize that the paucity of male Eu,*Dyrk1a*^+/*Osx-Cre*^ and Ts65Dn,*Dyrk1a*^+/+/*Osx-Cre*^ in all litters was most likely due to selective perinatal death. Dead pups found within litters were genotyped, but a Chi-square analysis indicated that observed genotype ratios of the dead pups were not different than the expected ratios, therefore, paucity of male genotypes may be the result of embryonic lethality. It was difficult to assess the contributions of *Dyrk1a* normalization in osteoblasts of male Ts65Dn animals, along with possible differences from Ts65Dn,*Dyrk1a*^+/+/+^ and Eu,*Dyrk1a*^+/+^ mice. 

### 4.5. Limitation: Potential off Target Effects of Osx-Cre Transgene

The *Osx-Cre* transgenic mice were originally generated by McMahon and obtained from Jackson Laboratories [43]. *Osterix* (*Osx*) is a zinc finger family transcription factor critical for osteoblast differentiation and both endochondral and intramembranous ossification [52,59,60,61]. During embryonic development *Osx* is expressed in the perichondrium and later in development expression is found in the periosteum and marrow cavity [43,59,62]. Previous *Osx-Cre* expression was found several other cell types including the olfactory bulb, GI tract, chondrocytes, stromal cells, perivascular cells, adipocytes, yet it is unclear if there is a potential influence of mediated gene deletion in these cell types on bone physiology [59,60]. *Osx-Cre* activated in the embryo targets additional cell types other than osteoblast lineage cells in postnatal mice which might be the result of unintended recombination activity [59]. Moreover, it has been shown that *Osx-Cre* transgenic mice exhibit unexpected skeletal phenotypes such as delayed calvarial ossification and cortical development, impaired fracture healing, decreased body weight [60,63]. *Osx-cre* mice expressing Cre during embryonic development have delayed growth, characterized by lower body weight and smaller bones compared to wild type controls at 6 weeks of age. There was delayed cortical expansion, decreased bone accrual, periosteal circumference, and cortical thickness in skeletally immature *Osx-cre* mice; without any changes in trabecular bone. However, low body weight and cortical deficits were resolved by 12 weeks of age. The cortical phenotype seen in *Osx-cre* mice was attributed an indirect effect of low body weight, based on statistical analysis correcting for body weight, cortical parameters were not significantly different than wildtype controls. The mechanism related to reduced body weight is unknown and thought to be an indirect effect of non-osseus Cre transgene expression [63]. Skeletal deficits associated with *Osx-Cre* could muddle studies on osteoblast biology. It is unclear how these defects arise in *Osx-Cre* transgenic mice, possibly due to insertional effects of the transgene, unintended deletion or duplication of DNA, prokaryotic vector sequences disrupting normal gene expression in mammals, or interruptive effect of foreign BAC DNA [60]. It was suggested that to control for *Osx-Cre* skeletal deficits, *Osx-Cre* control mice must be used to compared with the conditional knock-out mice. 

In our study, we used euploid and trisomic mice without a floxed *Dyrk1a* allele and with *Osx-Cre*, along with animals without *Osx-Cre*, as control animals for Eu,*Dyrk1a*^+/*Osx-Cre*^ and Ts65Dn,*Dyrk1a*^+/+/*Osx-Cre*^. Even though we had low numbers of mice in each group, we desired to see if there were potential differences in trabecular and cortical parameters for male and female Eu,*Dyrk1a*^wt/wt^ without *Osx-cre* expression versus Eu,*Dyrk1a*^wt/wt/*Osx-Cre*^ with *Osx-cre* expression; and Ts65Dn,*Dyrk1a*^wt/wt^ versus Ts65Dn,*Dyrk1a*^wt/wt/*Osx-Cre*^. There was no effect of *Osx-cre* in trabecular bone in either male and female euploid animals or female trisomic animals (Supplementary Material Table S1). Though were too few male trisomic animals to test, our data confirm previously reports of no effects of *Osx-cre* on trabecular bone. In cortical bone, there were deficits in CSA, Ct.Ar, Ct.Th, Ps.BS, Es.BS in female Ts65Dn,*Dyrk1a*^wt/wt/*Osx-Cre*^ mice compared to female trisomic controls, which were reflected in some mechanical parameters (Supplementary Material Tables S2 and S3). In male euploid mice, Eu,*Dyrk1a*^wt/wt/*Osx-Cre*^ had lower Ct.Ar and Ct.Th compared to wildtype controls. In addition to prior results showing the negative effects of *Osx-cre* on cortical bone, our data suggest that sex or ploidy augment these differences in cortical bone, especially before 12 weeks of age, but these assertations were limited by low sample sizes in male trisomic mice. 

## 5. Conclusions and Future Directions 

Trisomic *Dyrk1a* has been hypothesized to contribute to both cognitive impairment and skeletal development [48,64]. Previous results in our lab showed normalization of *Dyrk1a* copy number in all tissues from conception rescued DS-associated skeletal phenotypes in six-week-old Ts65Dn male mice [31]. Three copies of *Dyrk1a* have been hypothesized to be responsible for altered osteoblast and osteoclast activity and/or number leading to an imbalance in bone homeostasis [25,31]. Thus, *Dyrk1a* may be a potential target for therapeutic intervention in abnormal bone development in DS mouse models and humans with DS.

In this study we hypothesized that skeletal deficits associated with DS arise from increased gene dosage of *Dyrk1a* in osteoblasts and that normalization of functional *Dyrk1a* copy number in trisomic osteoblasts would lead to an improvement in skeletal phenotypes during development. Our results showed that *Dyrk1a* reduction in osteoblasts did not improve skeletal deficits in Ts65Dn,*Dyrk1a*^+/+/*Osx-Cre*^ as compared to Ts65Dn,*Dyrk1a*^+/+/+^ mice, and did not reach euploid levels, suggesting that trisomic *Dyrk1a* does not play a cell autonomous role in cellular function of osteoblasts cells. Therefore, skeletal abnormalities related to *Dyrk1a* may be the result of alternative molecular signaling pathways or communication between other cell types or tissues. Additionally, *Dyrk1a* reduction in both Eu,*Dyrk1a*^+/*Osx-Cre*^ and Ts65Dn,*Dyrk1a*^+/+/*Osx-Cre*^ led to increased skeletal deficits in female mice for total CSA, Ct.Ar, Ct.Th, Ps.BS, ultimate force, displacement to yield, and stiffness. These data suggest that *Dyrk1a* may need to maintain a stochiometric genetic balance for normal skeletal development and does not account for the origin of all skeletal anomalies seen in DS. 

Future studies will seek to identify how *Dyr1ka* expression alters signaling pathways related to osteoblast, osteoclast and osteocyte differentiation, function, and activity that results in abnormal skeletal phenotypes related to DS. We also strive to develop a methodology to determine if an interaction between modifier genes (disomic or trisomic) and trisomic *Dyrk1a* during critical timepoints of development could highlight novel developmental pathways associated with skeletal growth, development, and homeostasis. It is important to define critical spatiotemporal developmental periods when *Dyrk1a* is overexpressed to determine if normalization would rescue abnormalities associated with DS. Further investigations must identify cellular or molecular mechanisms that are disrupted by *Dyrk1a* kinase activity and how that contributes the bone abnormalities, then elucidate specific contributions of the targeted mechanisms to specific skeletal abnormalities.

## Figures and Tables

**Table 1:** Mechanical testing for 6-week-old female euploid and trisomic Ts65Dn mice.

	Eu,*Dyrk1a*^+*/+*^	Eu,*Dyrk1a*^+/*Osx-Cre*^	Ts65Dn,*Dyrk1a*^+/+/+^	Ts65Dn,*Dyrk1a^+/+/Osx-Cre^*	*p*-Value		
Female	(*n* = 20)	(*n* = 11)	(*n* = 9)	(*n* = 10)	*Dyrk1a* Normalization	Ploidy	*Dyrk1a* Normalization * Ploidy
Yield Force (N) ^a,b,^*	6.63 (0.36)	5.80 (0.14)	5.10 (0.87)	5.25 (0.45)	0.5106	**0.0455**	0.3327
**Ultimate Force (N) ^b,&^**	11.78 (0.46)	9.15 (0.19)	9.19 (0.62)	9.97 (0.63)	0.1032	0.1191	**0.0035**
**Displacement to Yield (µm) ^a,&^**	106.16 (4.14)	153.50 (9.50)	113.69 (15.05)	101.72 (3.73)	0.1384	0.0655	**0.0149**
Postyield Displacement (µm) ^a,^*	978.55 (84.18)	1376.44 (155.13)	663.44 (117.96)	935.20 (130.64)	0.0776	**0.0473**	0.7354
Total Displacment (µm) ^a,^*	1084.71 (83.85)	1529.94 (161.00)	777.14 (107.12)	1036.92 (131.25)	0.0694	**0.0402**	0.6271
**Stiffness (N/mm) ^&^**	67.62 (2.57)	45.77 (2.01)	47.73 (3.79)	55.83 (3.41)	0.0614	0.1774	**0.0001**
**Work to Yield (mJ) ^a^**	0.38 (0.04)	0.50 (0.04)	0.37 (0.13)	0.29 (0.03)	0.7784	0.1165	0.1805
Postyield Work (mJ) ^b,^*	8.18 (0.61)	7.07 (0.50)	4.64 (0.52)	6.35 (0.68)	0.7095	**0.0109**	0.0845
**Total Work (mJ) ^b^**	8.56 (0.62)	7.57 (0.52)	5.00 (0.45)	6.64 (0.70)	0.6959	**0.0085**	0.1143
**Yield Stress (MPa) ^a^**	82.19 (2.20)	87.97 (2.76)	79.15 (9.48)	78.71 (5.91)	0.6257	0.2647	0.5704
**Ultimate Stress (MPa)**	147.48 (3.37)	137.67 (2.74)	147.94 (5.91)	149.29 (5.75)	0.4145	0.2453	0.2828
**Strain to Yield (µε) ^a^**	28,589 (1277)	35,512 (1962)	28,788 (4329)	25,495 (932)	0.5195	0.0859	0.0743
Total Strain (µε) ^a,^*	291,841 (23,106)	358,541 (35,955)	191,803 (24,831)	255,989 (29,761)	0.1438	**0.0259**	0.9773
**Modulus (GPa)**	3.17 (0.09)	2.86 (0.09)	3.08 (0.16)	3.35 (0.16)	0.9107	0.1822	0.0583
**Resilience (MPa) ^a,b^**	1.27 (0.09)	1.76 (0.14)	1.40 (0.42)	1.08 (0.12)	0.6964	0.2334	0.0807
**Toughness (MPa) ^a^**	28.97 (1.94)	26.54 (1.62)	20.84 (2.37)	25.36 (2.85)	0.0745	0.0973	0.2127

In the property column, when males and females were analyzed together via 2-way ANOVA, when there was a main effect of ploidy, ‘^a^’ indicates a difference between euploid and trisomic animals (grouping Eu,*Dyrk1a*^+/+^ and Eu,*Dyrk1a*^+/*Osx-Cre*^ versus Ts65Dn,*Dyrk1a*^+/+/+^ and Ts65Dn,*Dyrk1a*^+/+/*Osx-Cre*^) and when there was a main effect of sex, ‘^b^’ indicates a difference between males versus females (*p* < 0.05). Post-hoc analysis. Males were examined alone via 2-way ANOVA (last column statistics) evaluated the effect of ploidy and *Dyrk1a* normalization in osteoblasts. When there was a main effect of ploidy, ‘*’ indicates difference between trisomic and euploid mice (*p* < 0.05). When the effect of *Dyrk1a* was examined, ‘^&^’ indicated an interactive effect (*p* < 0.05) between ploidy and *Dyrk1a*. Numbers that are bolded indicate significant *p* values.

**Table 2:** Mechanical testing for 6-week-old male euploid and trisomic Ts65Dn mice.

	Eu,*Dyrk1a^+/+^*	Eu,*Dyrk1a^+/Osx-Cre^*	Ts65Dn,*Dyrk1a^+/+/+^*	Ts65Dn,*Dyrk1a^+/+/Osx-Cre^*	*p*-Value		
Male	(*n* = 16)	(*n* = 7)	(*n* = 13)	(*n* = 8)	*Dyrk1a* Normalization	Ploidy	*Dyrk1a* Normalization * Ploidy
Yield Force (N) ^a,b,^*	7.95 (0.74)	8.32 (1.33)	7.21 (0.92)	5.56 (1.06)	0.1016	**0.0471**	0.4843
Ultimate Force (N) ^b,^*	12.45 (0.52)	11.54 (1.31)	10.33 (0.74)	9.56 (1.24)	0.3476	**0.0267**	0.9329
**Displacement to Yield (µm) ^a^**	187.06 (23.34)	245.07 (47.74)	231.57 (42.75)	149.73 (18.03)	0.2032	0.5269	0.1982
**Postyield Displacement (µm) ^a^**	690.33 (116.99)	385.26 (114.53)	577.24 (135.32)	783.98 (250.40)	0.8848	0.3856	0.1562
**Total Displacment (µm) ^a^**	877.40 (112.20)	630.33 (88.40)	808.81 (122.85)	933.71 (246.17)	0.6786	0.4344	0.2188
**Stiffness (N/mm)**	49.35 (3.46)	38.75 (8.39)	61.72 (25.69)	38.33 (3.81)	0.5931	0.1162	0.5161
**Work to Yield (mJ) ^a^**	0.90 (0.15)	1.21 (0.35)	1.09 (0.27)	0.47 (0.13)	0.2256	0.2385	0.2757
**Postyield Work (mJ) ^b^**	6.81 (0.93)	3.95 (1.16)	4.75 (0.94)	5.88 (1.49)	0.5343	0.9722	0.1374
**Total Work (mJ) ^b^**	7.71 (0.91)	5.16 (0.93)	5.84 (0.80)	6.36 (1.51)	0.3604	0.7713	0.1784
**Yield Stress (MPa) ^a^**	91.06 (9.01)	104.85 (14.81)	105.63 (13.18)	79.95 (11.57)	0.1264	0.6647	0.2141
**Ultimate Stress (MPa)**	140.68 (3.46)	140.90 (6.28)	146.48 (4.90)	140.53 (8.88)	0.6097	0.6287	0.5822
**Strain to Yield (µε) ^a^**	45,435 (5358)	56,656 (10,416)	52,890 (10,025)	32,576 (5181)	0.15	0.3139	0.1814
**Total Strain (µε) ^a^**	214,883 (27,918)	147,951 (20,679)	183,407 (25,559)	200,766 (51,810)	0.4693	0.7536	0.2239
**Modulus (GPa)**	2.27 (0.13)	1.87 (0.32)	3.46 (1.12)	2.72 (0.19)	0.3271	0.0605	0.3552
**Resilience (MPa) ^a,b^**	2.53 (0.43)	3.73 (1.06)	3.63 (0.87)	1.48 (0.40)	0.201	0.4356	0.1628
**Toughness (MPa) ^a^**	21.32 (2.47)	14.97 (2.63)	19.62 (3.12)	19.45 (4.04)	0.3274	0.6715	0.3618

In the property column, when males and females were analyzed together via 2-way ANOVA, when there was a main effect of ploidy, ‘^a^’ indicates a difference between euploid and trisomic animals (grouping Eu,*Dyrk1a*^+/+^ and Eu,*Dyrk1a*^+/*Osx-Cre*^ versus Ts65Dn,*Dyrk1a*^+/+/+^ and Ts65Dn,*Dyrk1a*^+/+/*Osx-Cre*^)and when there was a main effect of sex, ‘^b^’ indicates a difference between males versus females (*p* < 0.05). Post-hoc analysis. Males were examined alone via 2-way ANOVA (last column statistics) evaluated the effect of ploidy and *Dyrk1a* normalization in osteoblasts. When there was a main effect of ploidy, ‘*’ indicates difference between trisomic and euploid mice (*p* < 0.05). When the effect of *Dyrk1a* was examined. Numbers that are bolded indicate significant *p* values.

## Data Availability

The datasets used and/or analyzed during the current study are available from the corresponding author on reasonable request.

## Supplementary Materials

The following are available online at https://www.mdpi.com/article/10.3390/genes12111729/s1, Table S1: Potential effects of *Osx-Cre* in trabecular bone, Table S2: Potential effects of *Osx-Cre* in cortical bone, Table S3: Potential effects of *Osx-Cre* in mechanical parameters.
