# Supplementary material for: Skeletal Deficits in Male and Female down Syndrome Model Mice Arise Independent of Normalized Dyrk1a Expression in Osteoblasts
Source: Genes (Basel). 2021 Oct 28;12(11):1729. doi: 10.3390/genes12111729 (PMC8624983; doi:10.3390/genes12111729)
Supplement: Supplementary file 1 [file genes-12-01729-s001.zip › genes-1420462-supplementary.pdf]

Supplemental Table S1: Potential effects of *Osx-cre* in trabecular bone.

Female Ts65Dn Trabecular Bone	AVERAGE			
	Percent bone volume	Trabecular thickness	Trabecular separation	Trabecular number
Euploid,*Dyrk1a*$^{wt/wt}$, without Cre (n = 7)	24.66978	0.06931	0.172502857	3.527741429
Euploid,*Dyrk1a*$^{wt/wt}$, with Cre (n = 10)	25.92522	0.071803	0.173066	3.593003
TTEST	0.687523764	0.282163395	0.966286491	0.851648124
Trisomic, *Dyrk1a*$^{wt/wt}$, without Cre (n = 7)	23.61766429	0.068812857	0.189802857	3.34176
Trisomic, *Dyrk1a*$^{wt/wt}$, with Cre (n = 4)	18.918995	0.0643975	0.2075125	2.9536925
TTEST	0.435821224	0.206134615	0.643229452	0.592789407

Male Ts65Dn Trabecular Bone	Average			
	Percent bone volume	Trabecular thickness	Trabecular separation	Trabecular number
Euploid,*Dyrk1a*$^{wt/wt}$, without Cre (n = 3)	38.00312	0.07768	0.123517	4.888703
Euploid,*Dyrk1a*$^{wt/wt}$, with Cre (n = 6)	32.29261	0.079805	0.15032	3.975463
TTEST	0.3688	0.7452	0.1957	0.1321
	Percent bone volume	Trabecular thickness	Trabecular separation	Trabecular number
Trisomic, *Dyrk1a*$^{wt/wt}$, without Cre (n = 2)	21.63789	0.06913	0.18444	3.086785
Trisomic, *Dyrk1a*$^{wt/wt}$, with Cre (n = 1)	16.16818	0.06498	0.20155	2.48816

Supplemental Table S2: Potential effects of *Osx-cre* in cortical bone.

**Female Ts65Dn Cortical Bone**	**AVERAGE**					
	**Total CSA (mm^2)**	**Marrow Area (mm^2)**	**Cortical Area (mm^2)**	**Cortical Thickness (mm)**	**Periosteal BS (mm)**	**Endocortical BS (mm)**
**Euploid,*Dyrk1a*$^{wt/wt}$, without Cre (n = 7)**	1.487927225	0.758576568	0.729350657	0.197531852	5.142633937	3.806404556
**Euploid,*Dyrk1a*$^{wt/wt}$, with Cre (n = 10)**	1.394176879	0.715162401	0.679014478	0.189641532	4.993670831	3.674091913
**TTEST**	0.188586643	0.327784207	0.145894728	0.22764112	0.182866807	0.225663145
**Trisomic, *Dyrk1a*$^{wt/wt}$, without Cre (n = 7)**	1.385034276	0.694305233	0.690729043	0.19402097	5.014592042	3.663213009
**Trisomic, *Dyrk1a*$^{wt/wt}$, with Cre (n = 4)**	1.165305149	0.618837131	0.546468018	0.165553084	4.609058042	3.410917317
**TTEST**	**0.003190947**	0.118581785	**0.000856245**	**0.005145779**	**0.001164143**	**0.040335489**

**Male Ts65Dn Cortical Bone**	**AVERAGE**					
	**Total CSA (mm^2)**	**Marrow Area (mm^2)**	**Cortical Area (mm^2)**	**Cortical Thickness (mm)**	**Periosteal BS (mm)**	**Endocortical BS (mm)**
**Euploid,*Dyrk1a*$^{wt/wt}$, without Cre (n = 3)**	1.8408	0.9072	0.9336	0.2288	5.7052	4.1598
**Euploid,*Dyrk1a*$^{wt/wt}$, with Cre (n = 6)**	1.6099	0.8185	0.7914	0.2053	5.3143	3.8938
**TTEST**	0.1087	0.2865	**0.0461**	**0.0358**	0.0663	0.1894

	**AVERAGE**					
	**Total CSA (mm^2)**	**Marrow Area (mm^2)**	**Cortical Area (mm^2)**	**Cortical Thickness (mm)**	**Periosteal BS (mm)**	**Endocortical BS (mm)**
**Trisomic, *Dyrk1a*$^{wt/wt}$, without Cre (n = 2)**	1.4292	0.6746	0.7546	0.211	5.0925	3.5646
**Trisomic, *Dyrk1a*$^{wt/wt}$, with Cre (n = 1)**	1.0887	0.5323	0.5564	0.1765	4.4812	3.1757

Supplemental Table S3: Potential effects of *Osx-cre* in mechanical parameters.

**Female Mechanical Testing**	**AVERAGE**																	
	**I_ap (mm^4)**	**c_med (µm)**	**Yield Force (N)**	**Ultimate Force (N)**	**Displacement to Yield (µm)**	**Postyield Displacement (µm)**	**Total Displacment (µm)**	**Stiffness (N/mm)**	**Work to Yield (mJ)**	**Postyield Work (mJ)**	**Total Work (mJ)**	**Yield Stress (MPa)**	**Ultimate Stress (MPa)**	**Strain to Yield (µ?)**	**Total Strain (µ?)**	**Modulus (GPa)**	**Resilience (MPa)**	**Toughness (MPa)**
**Euploid,*Dyrk1a*$^{wt/wt}$,without Cre (n = 6)**	0.099302	805.1038667	6.687666667	11.39416667	113.0123333	1076.333333	1189.345667	63.556	0.4327	8.9532	9.3859	80.925	141.55	30535	318842	2.977	1.3705	31.039
**Euploid,*Dyrk1a*$^{wt/wt}$,with Cre (n = 7)**	0.087256714	777.7667286	6.066857143	10.92285714	103.3204429	939	1042.320443	64.124	0.3287	7.1869	7.5156	81.827	146.27	26745	270782	3.3232	1.1528	27.078
**TTEST**	0.28209725	0.28209725	0.281369349	0.494886693	0.643662933	0.438399096	0.512064086	0.4778	0.914	0.3543	0.1754	0.1586	0.8853	0.5735	0.3261	0.3888	0.1754	0.4508
**Trisomic, *Dyrk1a*$^{wt/wt}$,without Cre (n = 4)**	0.08351375	795.4505	7.433	10.91675	143.957725	419.75	563.707725	56.506	0.6291	4.1115	4.7405	104.9	156.47	38254	149203	3.0808	2.2898	18.269
**Trisomic, *Dyrk1a*$^{wt/wt}$,with Cre (n = 4)**	0.058926	697.7579	3.19225	7.62925	92.350325	768.25	860.600325	38.87	0.1611	4.5282	4.6893	58.695	139.68	21530	199393	3.0381	0.7002	20.531
**TTEST**	**0.037449058**	**0.037449058**	**4.16381E-05**	**0.009270851**	**0.002598123**	0.117142111	0.10892131	0.1309	**0.0238**	0.0989	0.6898	0.9539	**0.0081**	0.2356	0.0715	0.2343	0.9167	0.079
